# Supplementary material for: Concomitant Endoscopic Surgery for Bladder Tumors and Prostatic Obstruction: Are We Safely Hitting Two Birds with One Stone? A Systematic Review and Meta-Analysis
Source: J Clin Med. 2022 Oct 21;11(20):6208. doi: 10.3390/jcm11206208 (PMC9604964; doi:10.3390/jcm11206208)
Supplement: Supplementary file 1 [file jcm-11-06208-s001.zip › jcm-1945894-supplementary.pdf]

Data Supplement

Table of Contents

Supplementary Material S1: Pubmed search string 2

Supplementary Material S2: Newcastle – Ottawa Scale for cohort studies 3

Supplementary Material S3: References of all excluded studies with reasons for exclusion 5

Supplementary Material S4: Quality assessment of included studies 7

Supplementary Material S5: Sensitivity analysis with high-quality studies 10

Supplementary Material S6: Subgroup analysis based on single intravesical instillation of chemotherapy 11

Supplementary Material S7: Analysis of time to first recurrence 14

Supplementary Material S8: Publication bias assessment 15

Supplementary Material S9: Subgroup analysis of recurrence based on grade of bladder tumor and tumor's number. 16

Supplementary Material S10: Grading of evidence for all outcomes 18

Supplementary Material S1: Pubmed search string

("Benign Prostate Hyperplasia"[All Fields] OR "prostatic hyperplasia"[All Fields] OR "prostate"[All Fields]) AND ("simultaneous"[All Fields] OR "simultaneously"[All Fields] OR "coinstantaneous"[All Fields] OR "synchronous"[All Fields]) AND ("bladder cancer"[All Fields] OR "bladder tumor"[All Fields] OR "bladder tumor"[All Fields] OR "bladder neoplasm"[All Fields] OR "bladder carcinoma"[All Fields] OR "bladder carcinoma"[All Fields])

The search strategy was developed for PubMed and modified accordingly for the other databases.

Supplementary Material S2: Newcastle – Ottawa Scale for cohort studies

Selection

1) Representativeness of the exposed cohort

a) truly representative **(one star)**

b) somewhat representative **(one star)**

c) selected group

d) no description of the derivation of the cohort

2) Selection of the non exposed cohort

a) drawn from the same community as the exposed cohort **(one star)**

b) drawn from a different source

c) no description of the derivation of the non exposed cohort

3) Ascertainment of exposure

a) secure record (eg surgical records) **(one star)**

b) structured interview **(one star)**

c) written self report

d) no description

4) Demonstration that outcome of interest was not present at start of study

a) yes **(one star)**

b) no

Comparability (all two stars,two-three one star)

1) Comparability of cohorts on the basis of the design or analysis

a) study controls for bladder cancer recurrence **(one star)**

b) study controls for bladder neck prostatic urethra cancer recurrence **(one star)**

Outcome

1) Assessment of outcome

a) independent blind assessment **(one star)**

b) record linkage **(one star)**

c) self-report

d) no description

2) Was follow-up long enough for outcomes to occur

a) yes (select an adequate follow up period for outcome of interest) **(one star)**

b) no

3) Adequacy of follow up of cohorts

a) complete follow up - all subjects accounted for **(one star)**

b) subjects lost to follow up unlikely to introduce bias - small number lost less than 20% **(one star)**

c) follow up rate less than 80% and no description of those

d) no statement yes

Note: A study can be awarded a maximum of one star for each numbered item within the Selection and Outcome categories. A maximum of two stars can be given for Comparability.

Thresholds for quality assessment based on the Newcastle-Ottawa Scale for cohort studies

Good quality: 3 or 4 stars in selection domain AND 1 or 2 stars in comparability domain AND 2 or 3 stars in outcome/exposure domain

Moderate quality: 2 stars in selection domain AND 1 or 2 stars in comparability domain AND 2 or 3 stars in outcome/exposure domain

Poor quality: 0 or 1 star in selection domain OR 0 stars in comparability domain OR 0 or 1 stars in outcome/exposure domain

Supplementary Material S3: References of all excluded studies with reasons for exclusion

Overlapping studies

1. Castellani D, Gasparri L, Branchi A, Claudini R, Dellabella M. P42 - Concomitant transurethral resection of bladder tumor and prostate are oncological safe and improve quality of life: Results from a randomized controlled trial. Eur Urol Suppl. 2018;17(4):e2067.
2. Dellabella M, Castellani D, Gasparri L, Branchi A, Pavia M, Claudini R. P284 - Simultaneous transurethral resection of bladder tumor and prostate: Results from a randomized controlled trial. Eur Urol Suppl. 2018;17(8):357.
3. Ugurlu O, Gonulalan U, Adsan O, Kosan M, Oztekin V, Cetinkaya M. Effects of simultaneous transurethral resection of the prostate and solitary bladder tumours smaller than three centimetres on oncological results. Eur Urol Suppl. 2006;5(2):189.
4. Kim S, Park S, Kim S, Ahn H. MP-20.11: Oncologic Results of Simultaneous Transurethral Resection of Superficial Bladder Cancer and Benign Prostatic Hyperplasia. Urology. 2009;74(4, Supplement):S144.

No data for primary outcome

5. Giusti G, Caria N, Lucci Chiarissi M, De Vita G, Angelini L, Petrucci F, et al. Safety in simultaneous transurethral resection of bladder tumour and prostate. Eur Urol Suppl. 2019;18(9):e3245-e3246.

Case series

6. Chuang Hua Lee, Hwang T et al. Simultaneous transurethral resection of a bladder tumor and benign prostatic hyperplasia: Four case reports and literature review. Urol Sci. 2012;23(1):31–3.

Not releated to benign prostatic obstruction

7. B.F Schwartz et al. The role of cystoscopy before radical prostatectomy. Br J Urol. 1996;77(1):93–5.
8. Sofer M, Kaver I, Chen J, Nadu A, Beri A, Mabjeesh NJ, et al. Endourologic procedures combined with other surgical and urologic interventions--early experience. Urology. 2004 Nov;64(5):900–3.

No appropriate control arm

9. Sionov B V, Khunovich D, Benjamin S, Sidi AA, Tsivian A. 661 - Simultaneous transurethral resection of high grade bladder tumor and benign prostatic hyperplasia (BPH): Oncological safety. Eur Urol Suppl. 2017;16(3):e1149.
10. Tsivian A, Shtricker A, Sidi AA. Simultaneous transurethral resection of bladder tumor and benign prostatic hyperplasia: hazardous or a safe timesaver? J Urol. 2003 Dec;170(6 Pt 1):2241–3.
11. Glybochko P V, Alyaev YG, Pshikhachev AM, Sorokin NI, Dymov AM. [Choosing treatment for patients with bladder cancer combined with prostatic hyperplasia]. Urologiia. 2016 Nov;(5):92–6.

Editorial

12. Tsivian M, Tsivian A. Challenging the dogma of simultaneous resection of bladder tumor and

benign prostate. Vol. 7, Translational andrology and urology. 2018. p. S756–7.

Review

13. Kouriefs C, Loizides S, Mufti G. Simultaneous transurethral resection of bladder tumour and prostate: is it safe? Urol Int. 2008;81(2):125–8.

14. Chong T, Li Y, Wang Z, Shi T, Zhang P. [Simultaneous treatment for benign prostate hyperplasia and its concomitant diseases]. Zhonghua Nan Ke Xue. 2006 Jun;12(6):534–6.

Supplementary Material S4: Quality assessment of included studies

Supplementary Material S4.1: Overall risk of bias assessment based on the Newcastle-Ottawa Scale for cohort studies.

Study	Selection of cohorts				Comparability of cohorts	Outcome			Total score	Quality
	Representativeness of the exposed cohort	Selection of the non exposed cohort	Ascertainment of exposure	Demonstration that outcome of interest was not present at start of study	Comparability of cohorts on the basis of the design or analysis	Assessment of outcome	Was follow-up long enough for outcome to occur	Adequacy of follow up of cohorts		
Anastasopoulos. 2012			☆	☆	☆	☆	☆	☆	6	Moderate
Gargouri 2016			☆	☆	☆	☆	☆		5	Moderate
Greene 1972			☆	☆	☆	☆	☆		5	Moderate
Ham 2009		☆	☆	☆	☆☆	☆	☆	☆	8	Good
Iannucci 2005		☆	☆	☆		☆		☆	5	Poor

Jaidane 2010	☆	☆	☆	☆	☆☆	☆	☆	☆	9	Good
Kizilay 2020		☆	☆	☆	☆	☆	☆	☆	7	Good
Laor 1981		☆	☆	☆	☆	☆	☆		6	Good
Park 2009		☆	☆	☆	☆	☆	☆	☆	7	Good
Ugurlu 2007	☆	☆	☆	☆	☆	☆	☆	☆	8	Good
Vicente 1988		☆	☆	☆	☆	☆	☆	☆	7	Good
Wang 2020	☆	☆	☆	☆	☆☆	☆	☆	☆	9	Good

Supplementary Material S4.2: Overall risk of bias assessment according to the RoB 2 tool for RCTs.

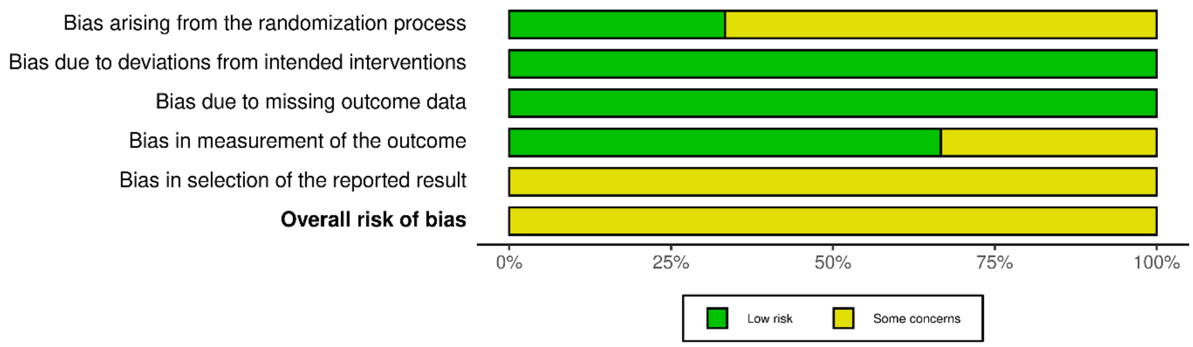

Data Supplement S4.3: Study-by-study risk of bias assessment according to the RoB 2 tool for RCTs.

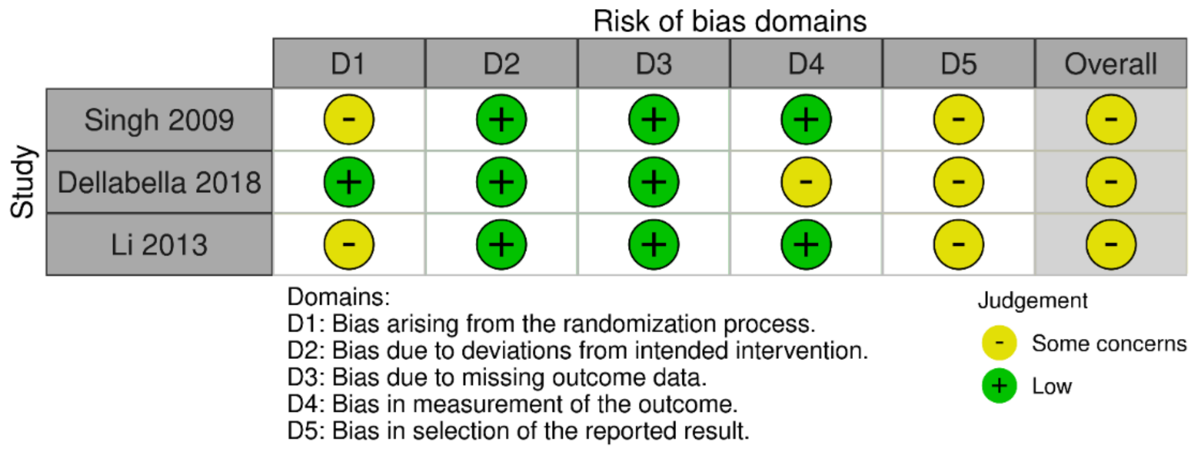

Supplementary Material S5: Sensitivity analysis with high-quality studies

Study	TURBT + TURP Events	TURBT + TURP Total	TURBT Events	TURBT Total	OR	95%-CI	Weight
Ham 2009	31	106	46	107	0.55	[0.31; 0.97]	14.6%
Jaidane 2010	17	85	20	85	0.81	[0.39; 1.69]	10.9%
Laor 1981	77	137	92	150	0.81	[0.51; 1.30]	17.4%
Park 2009	9	24	37	165	2.08	[0.84; 5.12]	8.1%
Ugurlu 2007	11	31	14	34	0.79	[0.29; 2.14]	6.9%
Vicente 1988	55	100	73	100	0.45	[0.25; 0.82]	14.0%
Wang 2020	32	118	38	118	0.78	[0.45; 1.37]	14.8%
Kizilay 2020	26	73	33	113	1.34	[0.72; 2.51]	13.1%
Heterogeneity: $I^2 = 42\%$, $p = 0.10$	**258**	**674**	**353**	**872**	**0.81**	**[0.60; 1.09]**	**100.0%**

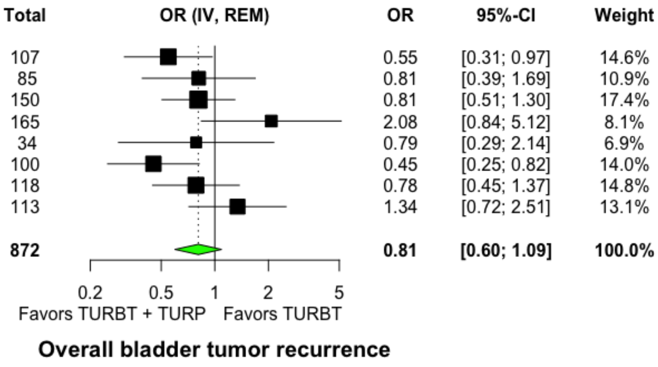

Data Supplement 5: Sensitivity analysis comparing TURBT and TURP versus TURBT in terms of overall bladder tumor recurrence. CI: confidence interval; IV: inverse variance; OR: odds ratio; RCT: randomized controlled trial; REM: random effects model; TURBT: transurethral resection of bladder tumor; TURP: transurethral resection of prostate.

Supplementary Material S6: Subgroup analysis based on single intravesical instillation of chemotherapy

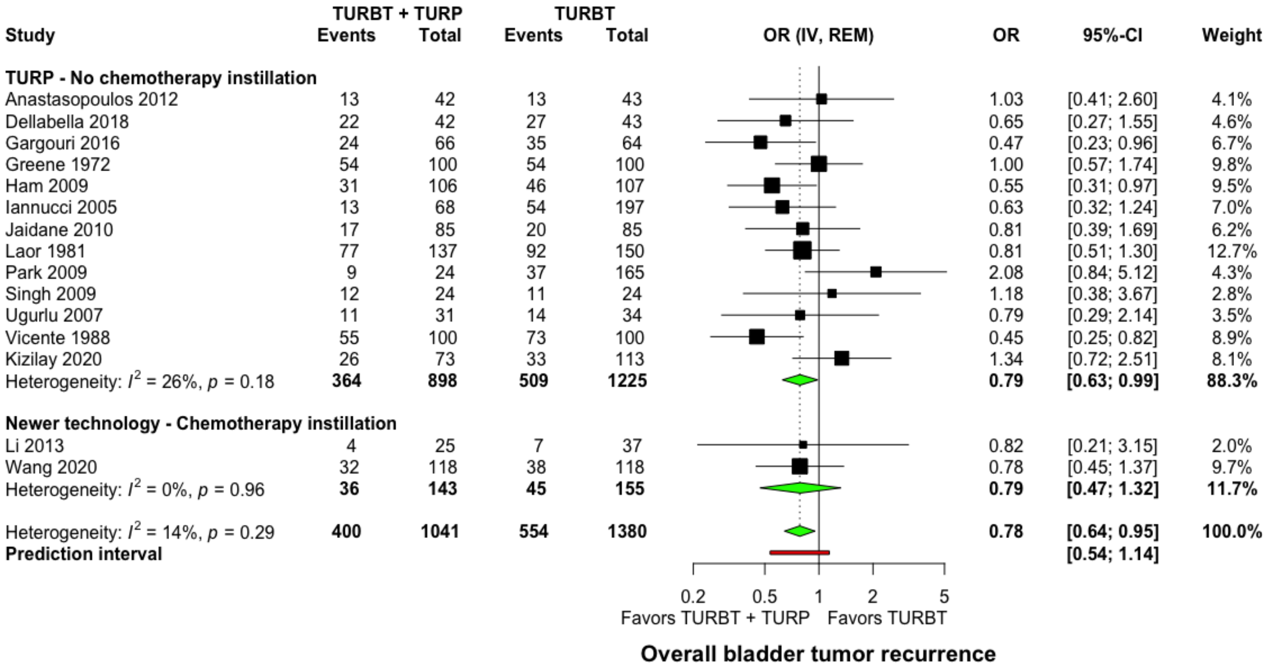

Data Supplement 6.A: Forest plot comparing TURBT and TURP versus TURBT in terms of recurrence based on the use of immediate intravesical chemotherapy instillation. CI: confidence interval; IV: inverse variance; OR: odds ratio; RCT: randomized controlled trial; REM: random effects model; TURBT: transurethral resection of bladder tumor; TURP: transurethral resection of prostate.

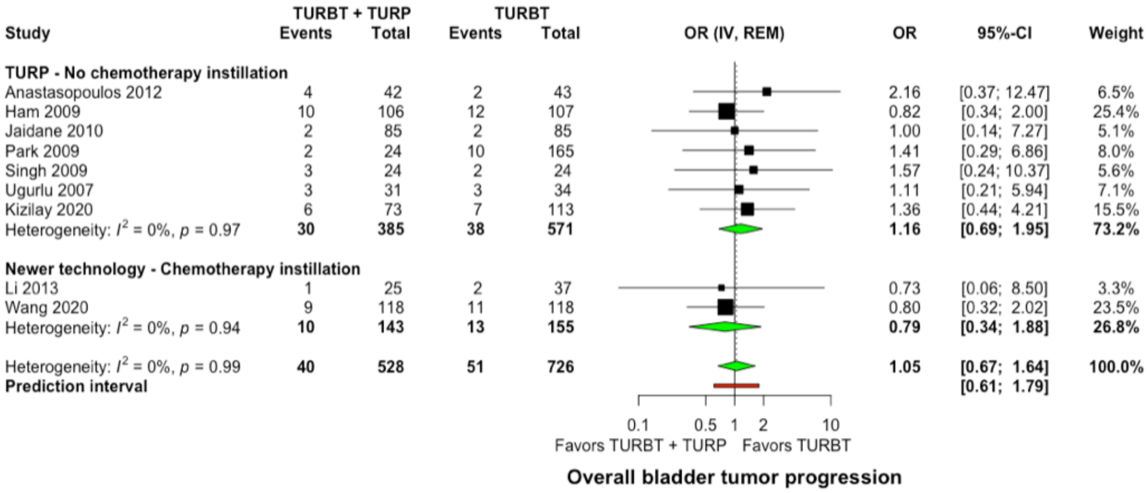

Data Supplement 6.B: Forest plot comparing TURBT and TURP versus TURBT in terms of tumor progression based on the use of immediate intravesical chemotherapy instillation. CI: confidence interval; IV: inverse variance; OR: odds ratio; RCT: randomized controlled trial; REM: random effects model; TURBT: transurethral resection of bladder tumor; TURP: transurethral resection of prostate.

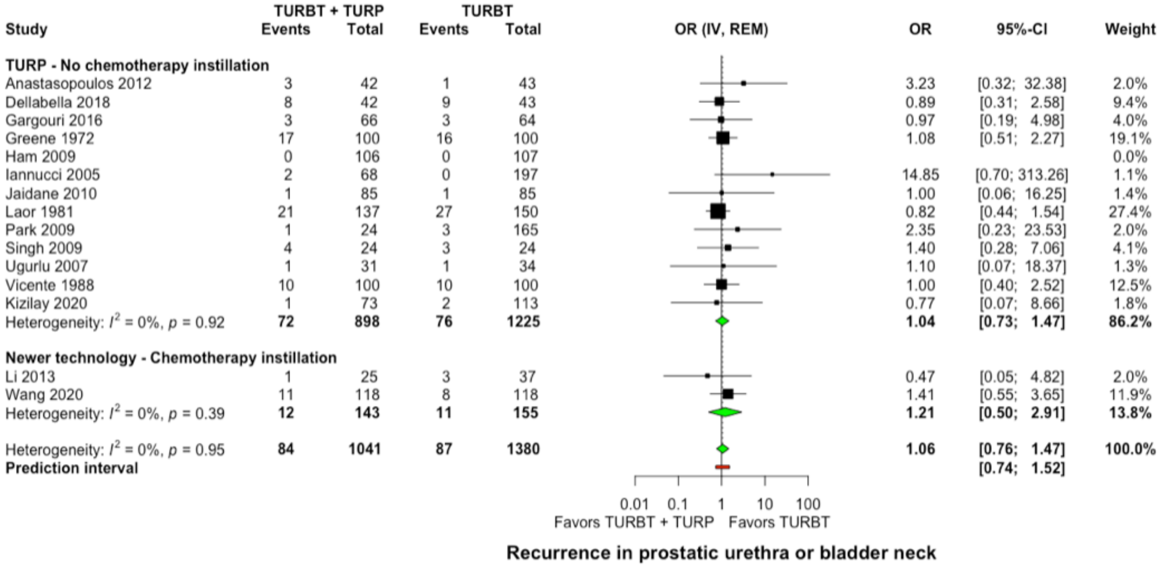

Data Supplement 6.C: Forest plot comparing TURBT and TURP versus TURBT in terms of tumor recurrence in prostatic urethra or bladder neck based on the use of immediate intravesical chemotherapy instillation. CI: confidence interval; IV: inverse variance; OR: odds ratio; RCT: randomized controlled trial; REM: random effects model; TURBT: transurethral resection of bladder tumor; TURP: transurethral resection of prostate.

Supplementary Material S7: Analysis of time to first recurrence

Study	TURBT + TURP Total	TURBT + TURP Mean	TURBT + TURP SD	TURBT Total	TURBT Mean	TURBT SD	MD	95%-CI	Weight
**RCT**									
Dellabella 2018	42	17.7	10.50	43	16.6	10.75	1.06	[-3.46; 5.58]	4.7%
Singh 2009	24	7.3	1.58	24	7.0	1.54	0.33	[-0.55; 1.21]	23.2%
Li 2013	25	13.5	3.50	37	11.6	3.20	1.90	[0.18; 3.62]	16.2%
Heterogeneity: $I^2 = 22\%$, $p = 0.28$	**91**			**104**			**0.82**	**[-0.24; 1.88]**	**44.1%**
**Non-RCT**									
Gargouri 2016	66	16.6	22.40	64	18.3	20.60	-1.70	[-9.09; 5.69]	2.0%
Laor 1981	137	28.0	18.66	150	31.0	3.83	-3.00	[-6.18; 0.18]	8.1%
Ugurlu 2007	31	20.2	16.18	34	13.7	6.65	6.50	[0.38; 12.62]	2.8%
Vicente 1988	100	14.0	16.50	100	16.0	12.00	-2.00	[-6.00; 2.00]	5.8%
Wang 2020	118	20.2	10.40	118	18.9	9.90	1.30	[-1.29; 3.89]	10.6%
Kizilay 2020	73	8.3	1.17	113	6.5	1.28	1.74	[1.38; 2.10]	26.6%
Heterogeneity: $I^2 = 66\%$, $p = 0.01$	**525**			**579**			**0.42**	**[-1.69; 2.53]**	**55.9%**
Heterogeneity: $I^2 = 65\%$, $p < 0.01$	**616**			**683**			**0.83**	**[-0.25; 1.90]**	**100.0%**
**Prediction interval**								**[-1.98; 3.63]**	

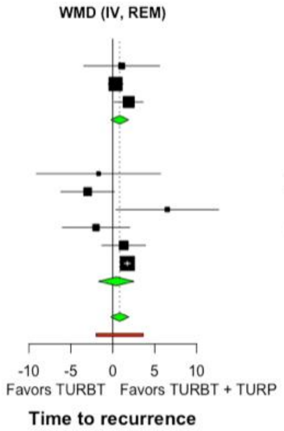

Data Supplement 7: Forest plot comparing TURBT and TURP versus TURBT in terms of time to first recurrence. CI: confidence interval; IV: inverse variance; MD: mean difference; RCT: randomized controlled trial; REM: random effects model; SD: standard deviation; TURBT: transurethral resection of bladder tumor; TURP: transurethral resection of prostate; WMD: weighted mean difference.

Supplementary Material S8: Publication bias assessment

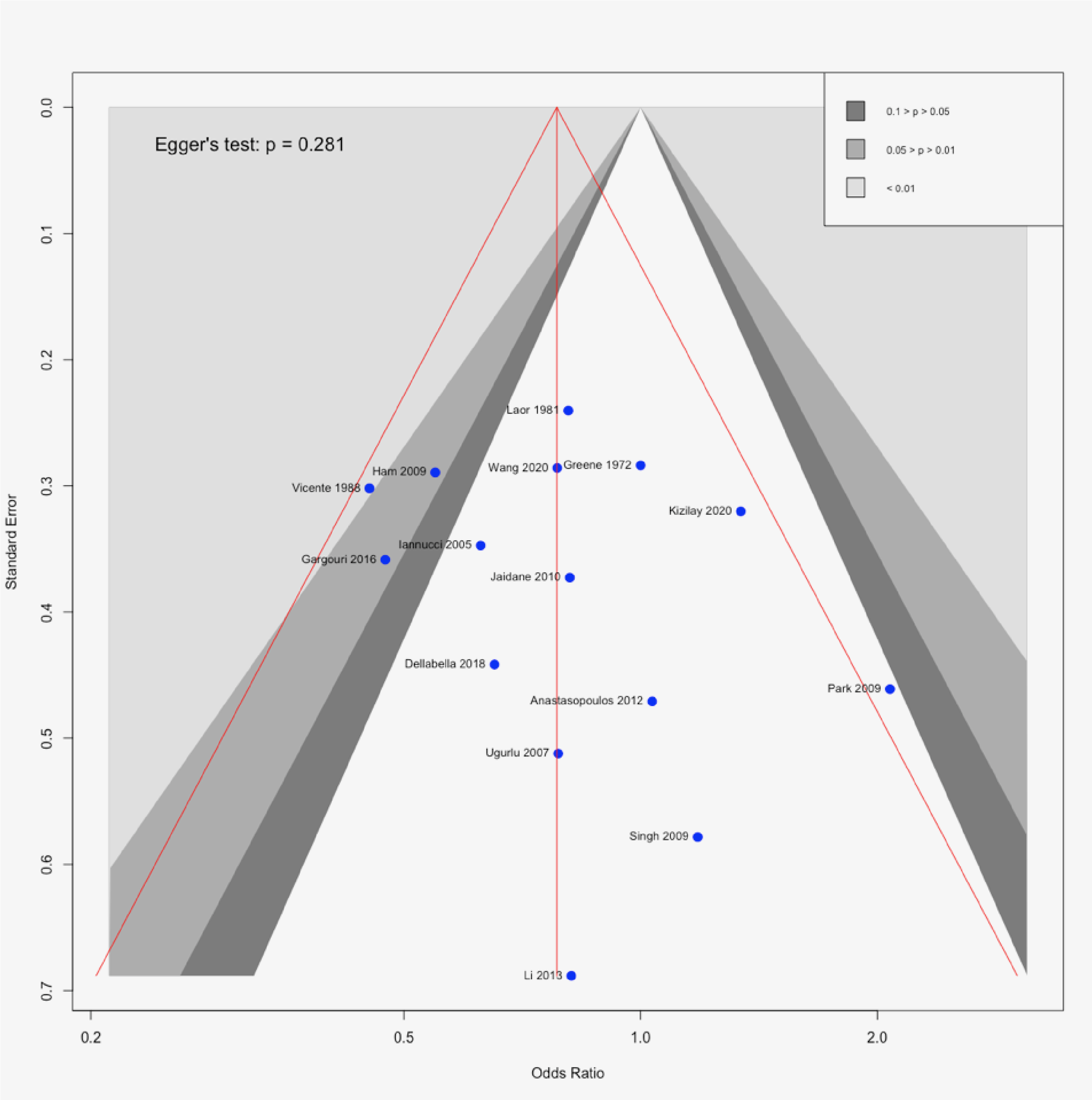

Data Supplement 8: Publication bias assessment with inspection of funnel plot asymmetry and Egger's statistical test.

Supplementary Material S9: Subgroup analysis of recurrence based on grade of bladder tumor and tumor's number.

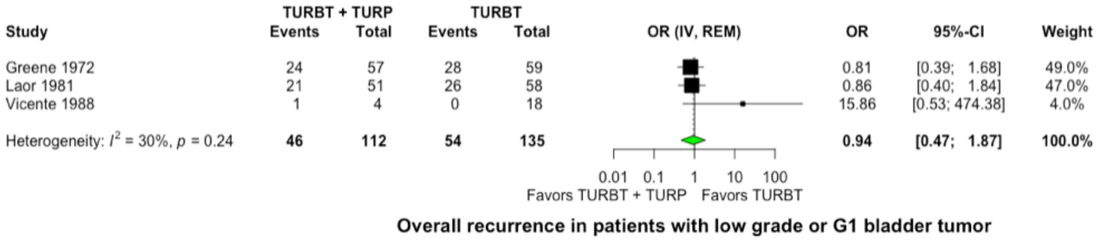

Data Supplement 9.A: Forest plot comparing TURBT and TURP versus TURBT in terms of low grade or G1 tumor recurrence. CI: confidence interval; IV: inverse variance; OR: odds ratio; RCT: randomized controlled trial; REM: random effects model; TURBT: transurethral resection of bladder tumor; TURP: transurethral resection of prostate.

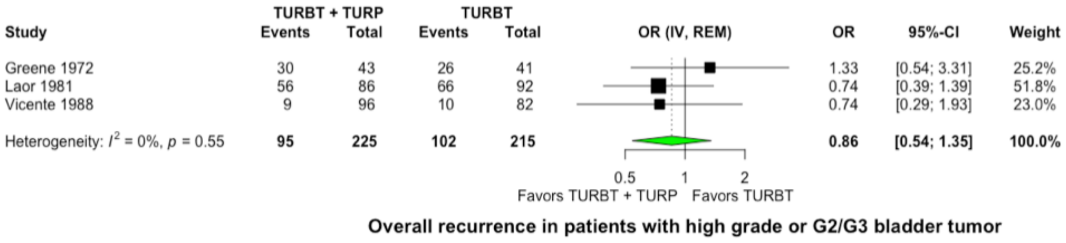

Data Supplement 9.B: Forest plot comparing TURBT and TURP versus TURBT in terms of high grade or G2/G3 tumor recurrence. CI: confidence interval; IV: inverse variance; OR: odds ratio; RCT: randomized controlled trial; REM: random effects model; TURBT: transurethral resection of bladder tumor; TURP: transurethral resection of prostate.

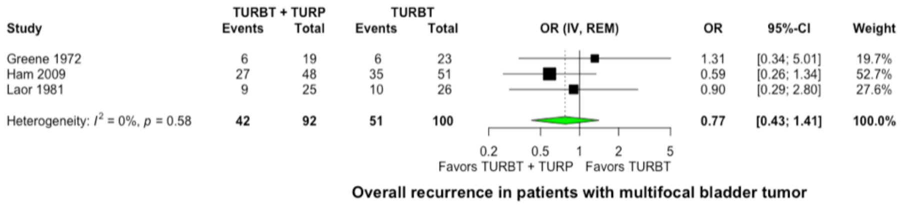

Data Supplement 9.C: Forest plot comparing TURBT and TURP versus TURBT in patients with multifocal tumor recurrence. CI: confidence interval; IV: inverse variance; OR: odds ratio; RCT: randomized controlled trial; REM: random effects model; TURBT: transurethral resection of bladder tumor; TURP: transurethral resection of prostate.

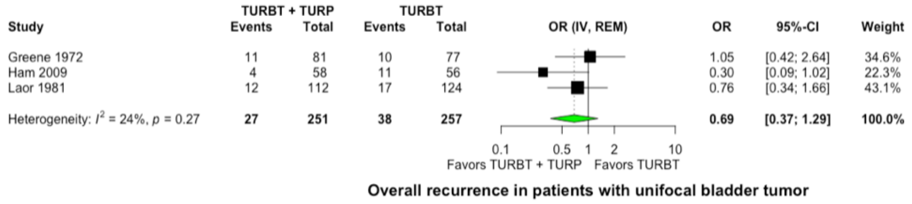

Data Supplement 9.D: Forest plot comparing TURBT and TURP versus TURBT in patients with unifocal tumor recurrence. CI: confidence interval; IV: inverse variance; OR: odds ratio; RCT: randomized controlled trial; REM: random effects model; TURBT: transurethral resection of bladder tumor; TURP: transurethral resection of prostate

Supplementary Material S10: Grading of evidence for all outcomes

Certainty assessment							№ of patients		Effect		Certainty	Importance
№ of studies	Study design	Risk of bias	Inconsistency	Indirectness	Imprecision	Other considerations	simultaneous transurethral resection of bladder tumor and prostate	transurethral resection of bladder tumor	Relative (95% CI)	Absolute (95% CI)		
**Overall bladder tumor recurrence**												
12	observational studies	serious [a]	not serious	not serious	not serious	all plausible residual confounding would suggest spurious effect, while no effect was observed	362/950 (38.1%)	509/1276 (39.9%)	**OR 0.78** (0.62 to 0.98)	**58 fewer per 1.000** (from 107 fewer to 5 fewer)	⨁⨁◯◯ LOW	CRITICAL
**Overall bladder tumor recurrence**												
3	randomized trials	very serious [b,c]	not serious	not serious	serious [d]	all plausible residual confounding would suggest spurious effect, while no effect was observed	38/91 (41.8%)	45/104 (43.3%)	**OR 0.81** (0.44 to 1.50)	**51 fewer per 1.000** (from 181 fewer to 101 more)	⨁⨁◯◯ LOW	CRITICAL
**Bladder neck/Prostatic Urethra recurrence**												
12	observational studies	serious [a]	not serious	not serious	not serious	all plausible residual confounding would suggest spurious effect, while no effect was observed	71/950 (7.5%)	72/1276 (5.6%)	**OR 1.09** (0.76 to 1.55)	**5 more per 1.000** (from 13 fewer to 28 more)	⨁⨁◯◯ LOW	CRITICAL
**Bladder neck/Prostatic Urethra recurrence**												
3	randomized trials	very serious [b,c]	not serious	not serious	serious [d]	all plausible residual confounding would suggest spurious effect, while no effect was observed	13/91 (14.3%)	15/104 (14.4%)	**OR 0.92** (0.40 to 2.12)	**10 fewer per 1.000** (from 81 fewer to 119 more)	⨁⨁◯◯ LOW	CRITICAL

Certainty assessment							№ of patients		Effect		Certainty	Importance
**№ of studies**	**Study design**	**Risk of bias**	**Inconsistency**	**Indirectness**	**Imprecision**	**Other considerations**	**simultaneous transurethral resection of bladder tumor and prostate**	**transurethral resection of bladder tumor**	**Relative (95% CI)**	**Absolute (95% CI)**		
**Bladder tumor progression**												
7	observational studies	serious [a]	not serious	not serious	not serious	all plausible residual confounding would suggest spurious effect, while no effect was observed	36/479 (7.5%)	47/665 (7.1%)	**OR 1.03** (0.65 to 1.65)	**2 more per 1.000** (from 24 fewer to 41 more)	⨁⨁◯◯ LOW	CRITICAL
**Bladder tumor progression**												
2	randomized trials	very serious [b,c]	not serious	not serious	serious [d]	all plausible residual confounding would suggest spurious effect, while no effect was observed	4/49 (8.2%)	4/61 (6.6%)	**OR 1.18** (0.26 to 5.28)	**11 more per 1.000** (from 48 fewer to 205 more)	⨁⨁◯◯ LOW	CRITICAL
**Time to recurrence**												
6	observational studies	serious [a]	not serious	not serious	not serious	all plausible residual confounding would suggest spurious effect, while no effect was observed	525	579	-	MD **0.42 months higher** (1.69 lower to 2.53 higher)	⨁⨁◯◯ LOW	CRITICAL
**Time to recurrence**												
3	randomized trials	very serious [b,c]	not serious	not serious	serious [d]	all plausible residual confounding would suggest spurious effect, while no effect was observed	91	104	-	MD **0.82 month higher** (0.24 lower to 1.88 higher)	⨁⨁◯◯ LOW	CRITICAL

CI: Confidence interval; **OR:** Odds ratio

a. Retrospective design, failure to adequately control confounding; b. Lack of allocation concealment; c. Lack of blinding; d. Small sample size.
